# Removal of Rust from Steel Instruments

**Published:** 1905-03

**Authors:** 


### REMOVAL OF RUST FROM STEEL INSTRUMENTS.

To remove rust from steel instruments, Sangu recommends placing them overnight in a saturated solution of zinc chloride. The rust disappears through reduction. On removing the instruments they are to be rinsed in clear water, placed in hot soda and soap solution and dried. It is also advantageous to polish with absolute alcohol and chalk.—*Central Medical Magazine.*

